# Hypoxic and Highly Angiogenic Non-Tumor Tissues Surrounding Hepatocellular Carcinoma: The ‘Niche’ of Endothelial Progenitor Cells

**DOI:** 10.3390/ijms11082901

**Published:** 2010-08-09

**Authors:** De-Cai Yu, Jun Chen, Yi-Tao Ding

**Affiliations:** 1 Institute of Hepatobiliary Surgery, The Affiliated Drum Tower Hospital, School of Medicine, Nanjing University, Nanjing, Jiangsu Province, China; E-Mail: dryudecai@hotmail.com; 2 Department of Hepatobiliary Surgery, The Affiliated Drum Tower Hospital, School of Medicine, Nanjing University, Nanjing, Jiangsu Province, China; E-Mail: dryudecai@hotmail.com; 3 Department of Pathology, The Affiliated Drum Tower Hospital, School of Medicine, Nanjing University, Nanjing, Jiangsu Province, China

**Keywords:** endothelial progenitor cells, hepatocellular carcinoma, liver cirrhosis, angiogenesis

## Abstract

Our previous investigations showed that mobilized endothelial progenitor cells (EPCs) are enriched in non-tumor tissues (NT) surrounding hepatocellular carcinoma (HCC), compared to in tumor tissues (TT). This particular recruitment of EPCs is worth investigating further. The mobilization, recruitment, homing, and incorporation of EPCs into tumors require the participation of multiple factors, including angiogenic factors, adherent molecules, endothelial cells, hypoxic environment, *etc*. Therefore, we hypothesized that NT might be a hypoxic and highly angiogenic area, into which many more EPCs are recruited and homed. In the last three years, we evaluated the hypoxic condition, angiogenic factors and angiogenic index using frozen tissues or tissue microarrays from 105 patients who had undergone hepatectomy for HCC, and here we review our results and the studies of others. All results showed the expression of Hypoxiainducible factor-1α was higher in NT than in TT. The expression of VEGFA, bFGF, TGF-β, MCP-1, MMP-9, TIMP-2, and endostatin in NT was significantly higher than in normal liver and TT. Meanwhile, the expression of CD105—the surface marker of activated endothelial cells—was also higher in NT than in TT at the protein and mRNA levels. These investigations showed that NT is a hypoxic and highly angiogenic area, which may be the ‘niche’ of EPCs. The particular background in HCC may be related to liver cirrhosis. Therefore, non-tumor tissues surrounding HCC may be the ‘niche’ of endothelial progenitor cells.

## 1. Introduction

It is generally accepted that tumors are endowed with angiogenic inducing capability, and tumor growth, invasion and metastasis are angiogenesis-dependent. Over the past several years, basic and clinical investigations suggested that neoangiogenesis involves bone marrow derived endothelial progenitor cells (EPCs) as well as endothelial cells (ECs) co-opted from surrounding vessels [1–3]. EPCs are mobilized, recruited, and homed with high specificity into solid tumors [4,5]. Therefore, EPCs may be useful to detect early tumors, distinguish benign from malignant disease, determine prognosis, predict the response to therapy, and monitor the clinical course, *etc.* [6–8].

Our previous investigations supported that mobilized EPCs participated in tumor vasculogenesis of hepatocellular carcinoma (HCC), and the phenotype of EPCs—CD133—could be used as a biomarker for predicting the progression of HCC. Moreover, that the levels of integrated EPCs in newly formed blood vessels had been reported to be as high as 16.56% in tumor tissues, 72.24% in adjacent non-tumor tissues, and 55.86% in tumor free tissues, according to the ratio of CD133-MVD(Microvessel Density) and CD34 MVD. EPCs were enriched in non-tumor tissues surrounding hepatocellular carcinoma (NT), and not in tumor tissues (TT) [9]. The molecular mechanism of the recruitment of much more EPCs into NT was not known.

The mobilization, recruitment, homing, and incorporation of EPCs into tumors is a multi-step and multi-factor event. This complicated process requires the participation of multiple factors, including angiogenic factors, adherent molecules, tumor cells, ECs, stromal cells, and a hypoxic environment [10]. Therefore, it was hypothesized that NT might be a hypoxic and highly angiogenic area, into which many more EPCs were recruited and homed. To test this hypothesis, we detected the hypoxic condition, angiogenic factors and angiogenic index within frozen tissues or tissue microarrays constructed as described previously [11], and here review our previous studies and others.

## 2. Non-Tumor Tissues Surrounding Hepatocellular Carcinoma: Hypoxic Area

Hypoxia-inducible factor-1 (HIF-1), composed of α and β subunits, is a pivotal regulator of the cellular response to hypoxia [12]. The HIF-1α subunit becomes stabilized or even induced in response to hypoxia [13]. HIF-1α is highly expressed in HCC specimens, and significantly correlated with venous invasion and lymph node invasion [14]. The disease-free survival time of patients with high HIF-1α expression was significantly shorter than that of the low expression group [15]. Our previous results showed the expression of HIF-1α in NT was higher than in TT by immunohistochemistry and Western blotting analysis [16]. Therefore, NT might be a hypoxic area. Of note, HIF-1α is an important transcription factor of lots of angiogenic factors, which are detected to check the contradiction in the further studies.

## 3. Non-Tumor Tissues Surrounding Hepatocellular Carcinoma: High-Level Expression of Angiogenic Factors

We have further evaluated the expression of some major angiogenic factors in NT and TT with tissue arrays, such as activator molecules (vascular endothelial growth factor 165, VEGFA; basic fibroblast growth factor, bFGF; transforming growth factor-β, TGF-β; monocyte chemoattractant protein-1, MCP-1; metal metalloproteinase-9, MMP-9), inhibitor molecules (thrombospondin-1, TSP-1; endostatin; tissue inhibitors of metalloproteinase 1 and 2, TIMP-1 and TIMP-2), and corresponding transcript factors (cyclooxygenase-2, COX-2; inducible nitric oxide synthase, NOS-2). The immunoreactivity of VEGFA, bFGF, TGF-β, MCP-1, TSP-1, TIMP-1, TIMP-2, and endostatin was observed mainly in the tumor and non-tumor hepatic cells, showing a predominant cytoplasmic staining, with the positive liver cells distributed in both the tumor tissue and surrounding liver. Cytoplasmic and nuclear staining for COX-2 and NOS-2 was also observed both in the tumor and non-tumor hepatic cells. The expression of VEGFA, bFGF, TGF-β, MCP-1, TSP-1, MMP-9, TIMP-2, and endostatin was significantly higher in NT than that in normal liver and TT (P < 0.01 or 0.05), while no significant difference was found in TIMP-1, COX-2, and NOS-2 between TT and NT. Meanwhile, VEGFA, bFGF, TGF-β, MCP-1, TSP-1, MMP-9, TIMP-2, and endostatin were also constitutively expressed in normal liver tissue, but with a lower expression level than in NT or TT.

More and more investigations also reported that proangiogenic factors, such as VEGFA [17,18], hepatic growth factor (HGF, refer to [19]), and NOS-2 [20], have higher expression in the liver tissues surrounding HCC than in tumors. Moreover, macrophage colony-stimulating factors (M-CSF) and counts of macrophages were higher in peritumoral liver tissue than in tumor tissue [21], as reported previously by others [22]. Of note, inhibitors were also up-regulated with angiogenic activators.

Therefore, non-tumor tissues surrounding HCC is a hypoxic area with high expression of angiogenic factors, which is different from non-tumor tissues surrounding other tumors.

## 4. Non-Tumor Tissues Surrounding Hepatocellular Carcinoma: A Highly Angiogenic Area

Endoglin (CD105) is a homodimeric transmembrane glycoprotein highly expressed on activated endothelial cells, and is involved in vascular development and remodeling [23]. Compared to the conventional biomarker CD34, CD105 has been demonstrated to be a superior angiogenesis marker in breast cancer, malignant melanoma, non-small cell lung cancer, and colorectal carcinoma [24–27].

We previously demonstrated the superiority of CD105 to CD34 as a marker of angiogenesis in HCC [11], which was consistent with the investigation of Ho *et al.* [28]. Our further studies revealed that CD105 was positively stained mostly in a subset of microvessels ‘endothelial sprouts’ in TT of all patients while CD105 showed diffuse positive staining, predominantly on hepatic sinus endothelial cells in the surrounding of draining veins in NT. Using a paired *t* test, the expression of CD105 in NT was higher than in TT at both the protein and mRNA levels. It is demonstrated that CD105 was not only present in neovessels in TT, but also more abundant in hepatic sinusoidal endothelial cells in non-tumor tissues (NT) with cirrhosis. Therefore, it was concluded that there are more activated endothelial cells in NT, which was a highly angiogenic area.

## 5. Non-Tumor Tissues Surrounding Hepatocellular Carcinoma: The ‘Niche’ of Endothelial Progenitor Cells

It is clear that the mobilization, recruitment, homing, and incorporation of EPCs into tumors requires the participation of multiple factors, including angiogenic factors, adherent molecules, tumor cells, ECs, stromal cells, and a hypoxic environment [29]. All previous mentioned results showed that NT is a hypoxic and highly angiogenic area with a high level of EPCs [9,16,29]. Reasonably, NT might be the ‘niche’ of EPCs.

An increase in tumor mass during tumor growth leads to a hypoxic environment, which results in the production of pro-angiogenic growth factors; the onset of the angiogenesis switch [30]. The pro-angiogenic factors, such as VEGFA, bFGF, and MCP-1, *etc.*, are involved in the activation, mobilization and recruitment of EPCs from the bone marrow, and the differentiation of EPCs into ECs in some ischemic diseases and during tumor growth [31,32]. Of note, many more EPCs were mobilized into the circulation in the patient group with unresectable HCC compared to the patient group with resectable HCC and liver cirrhosis, and positively correlated with plasma VEGF, IL-8, and AFP levels [9,33]. Furthermore, these factors activate MMPs, particularly MMP-9, which lead to the release of soluble KIT ligand, in turn promoting cell proliferation and motility within the bone marrow microenvironment [34]. In NT, there was higher expression of many angiogenic factors, and much more EPCs were recruited into these areas [9]. Furthermore, in the NT there were more active ECs, which may recruit EPC as components of the tumor-induced stroma. Therefore, NT might provide a particular microenvironment for EPCs to home.

## 6. Particular Background in HCC: Liver Cirrhosis

In general, HCC is a cancer that is associated in most cases with chronic liver disease, such as chronic viral hepatitis and cirrhosis, especially in Southeast Asia. The nonmalignant liver itself has a precancerous change with angiogenesis. During liver cirrhosis, fibrogenesis induces intrahepatic shunts and a barrier between the sinusoids and the hepatocytes [35], where hypoxia appears. Fibrous pseudo lobes form as a discrete hypoxia unit to induce angiogenesis [36]. Furthermore, hepatitis B virus X protein increases the transcriptional activity and protein level of HIF-1α, and thereby promotes angiogenesis during hepatocarcinogenesis [37]. Therefore, the cells in cirrhotic liver are under a sustained, mechanically reduced blood flow, which induces angiogenesis in cirrhotic tissues [38]. Sieghart *et al.* reported that circulating EPC are increased in patients with portal hypertension +/− hepatocellular carcinoma. The negative correlation of EPC with hepatic venous pressure gradient suggests a protective role of EPC in liver cirrhosis, whilst vascular endothelial growth factor is associated with high hepatic venous pressure gradient [39,40]. Consistently, EPCs were mobilized and recruited into cirrhotic tissue, and incorporated into neovessels during liver cirrhosis [41]. Moreover, transplanted EPC ameliorate carbon tetrachloride-induced liver cirrhosis in rats [42]. Of note, human peripheral blood EPC transplantation significantly enhanced vascularization and improved survival rates after acute liver injury in mice [43]. The particular pathology in HCC with liver cirrhosis may be related with distribution, contribution, origin, and differentiation of EPCs, and EPCs might be regarded as targeting vectors for therapy and diagnosis in liver disease.

Therefore, NT was a hypoxic area rich in angiogenic factors and highly angiogenic conditions, which may be a reflection of both cirrhosis and a “field effect” relevant to the tumor. In summary, the recruitment and home of EPCs into NT might be related to the particular microenvironments in HCC with liver cirrhosis.

## 7. Perspective

In summary, we found that the high expression of angiogenic factors in NT, but not in TT, is compatible with the findings of active ECs and EPCs numbers. These microenvironments in TT and NT might play important roles in the progression of HCC. Of note, high incidence of intrahepatic metastasis and recurrence after resection suggested that the micro-environment in NT was an important but often neglected issue. Together with these results, it was concluded that postoperative adjuvant therapies should target not only the residual tumor cells, but also the ‘soil’, such as NT, which provides a microenvironment for tumor metastasis and growth. Therefore, we propose that the microenvironment in NT with high angiogenesis was also very important for understanding the mechanism of intrahepatic metastasis of HCC and in shaping the postoperative strategy for prevention of recurrence after hepatectomy. Moreover, EPCs may become new targets of adjuvant therapy, which await further investigation in the future.
